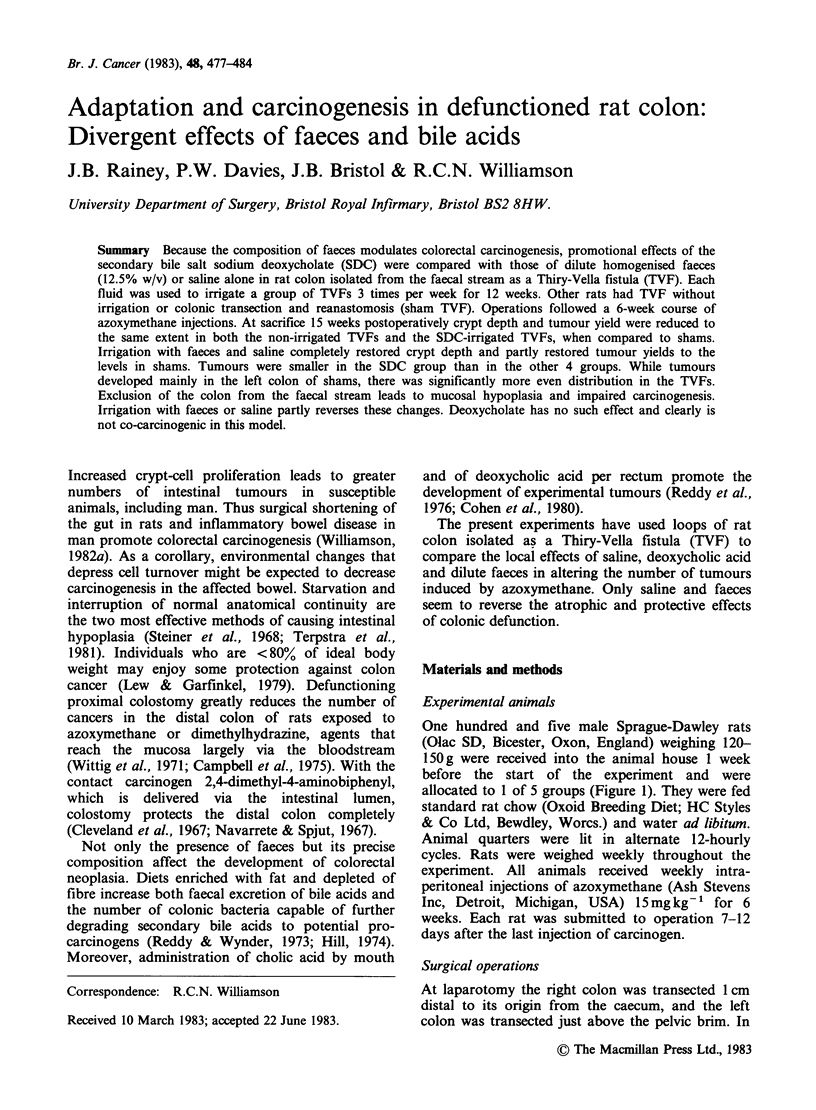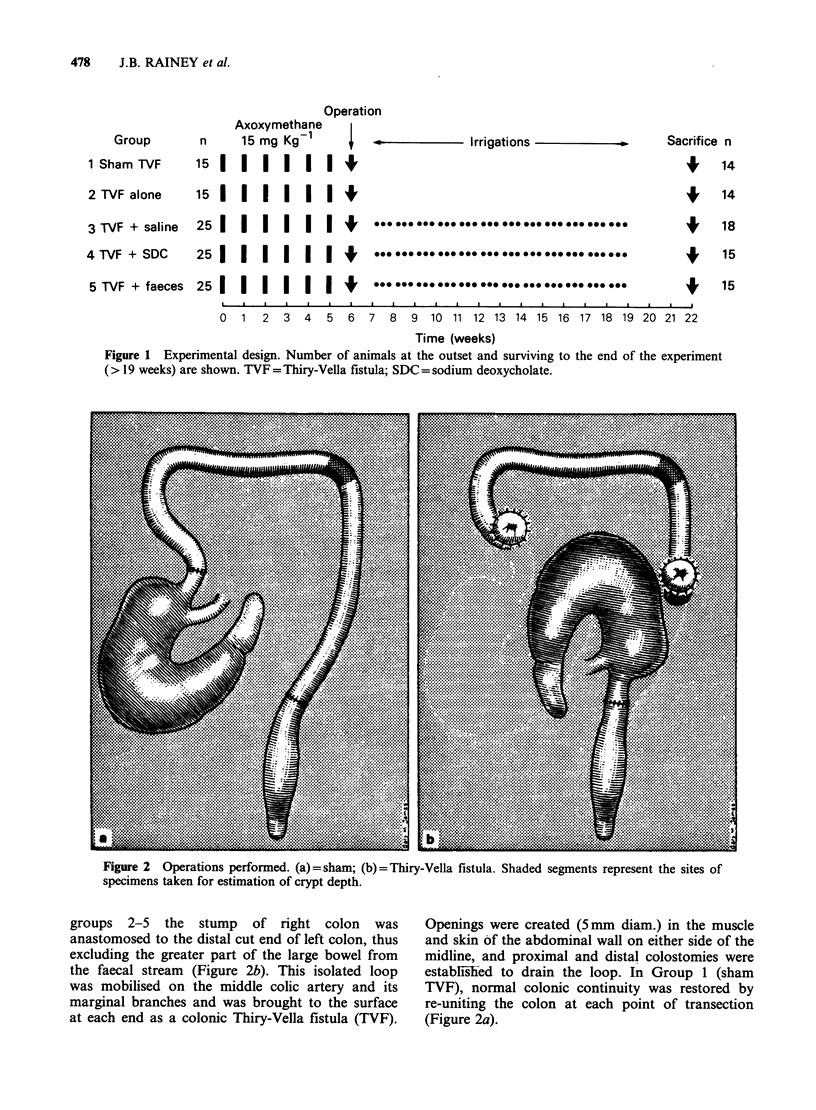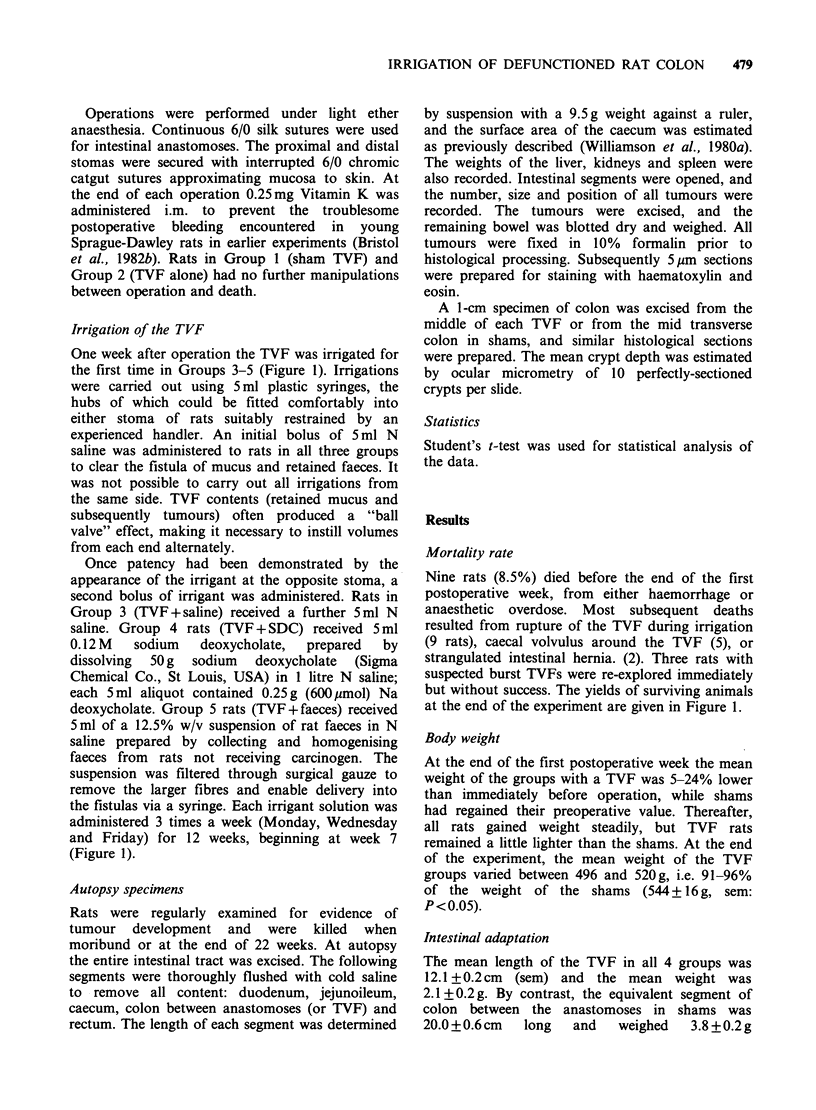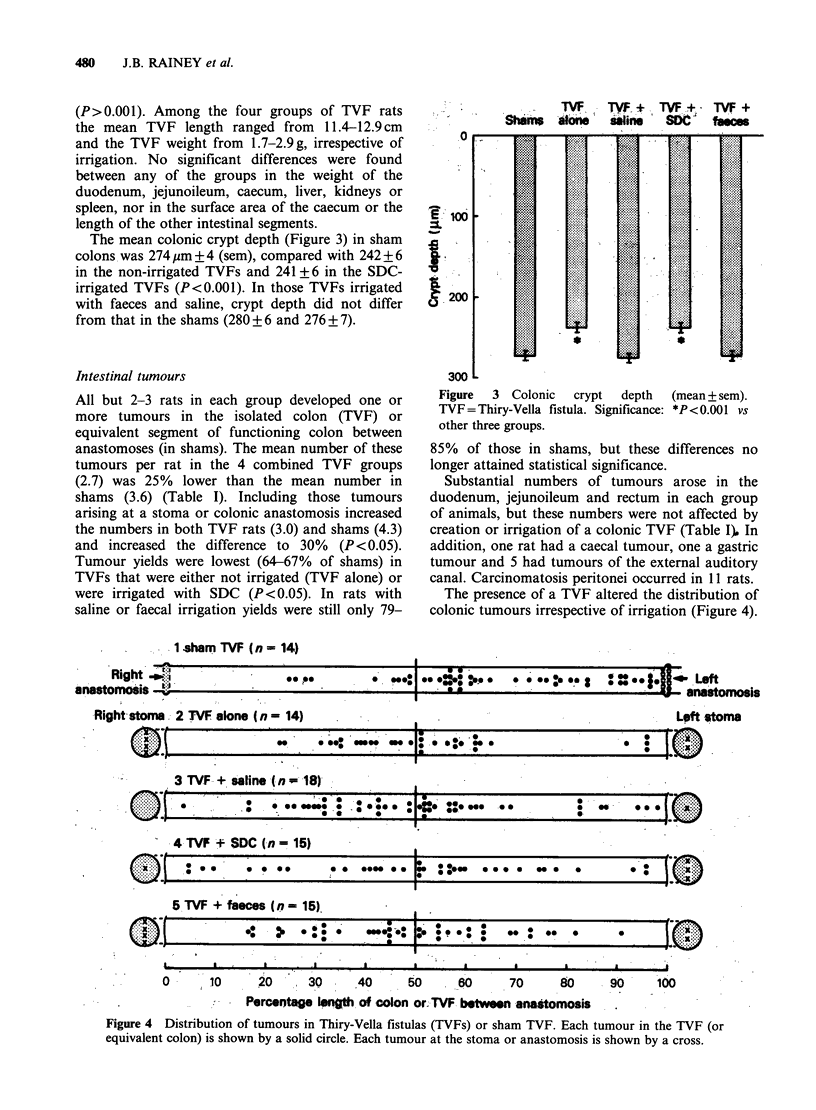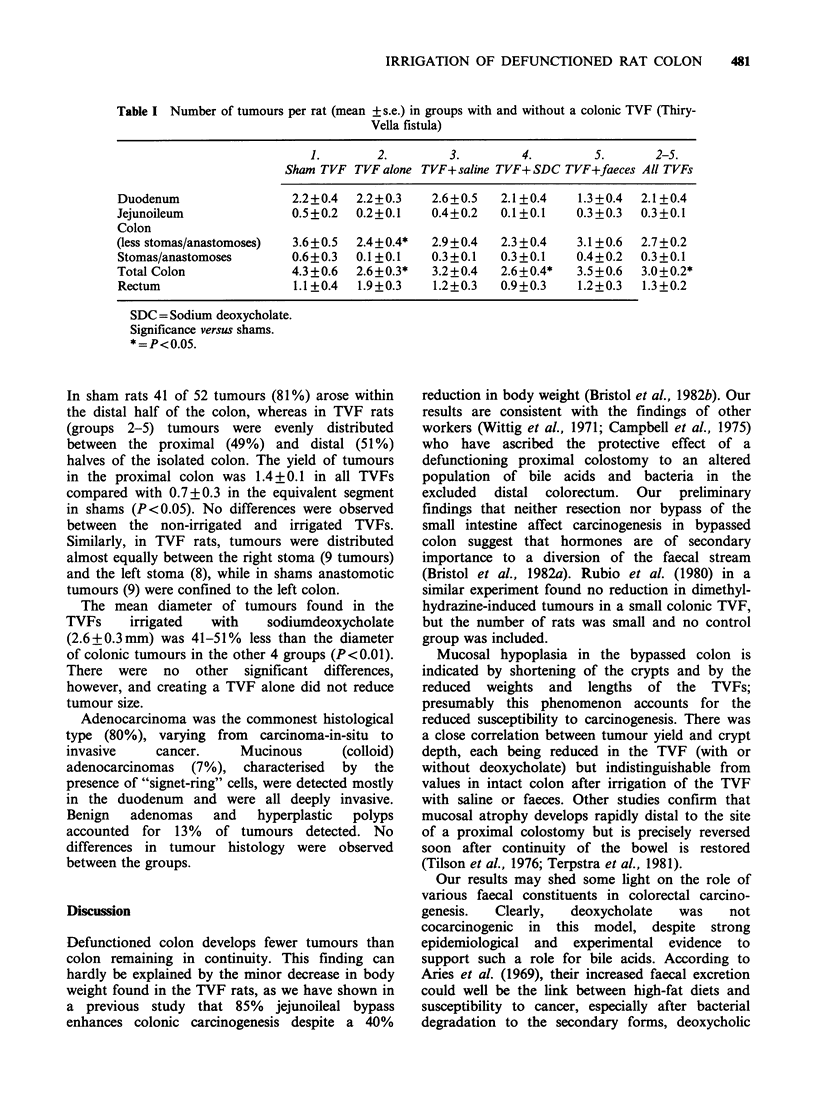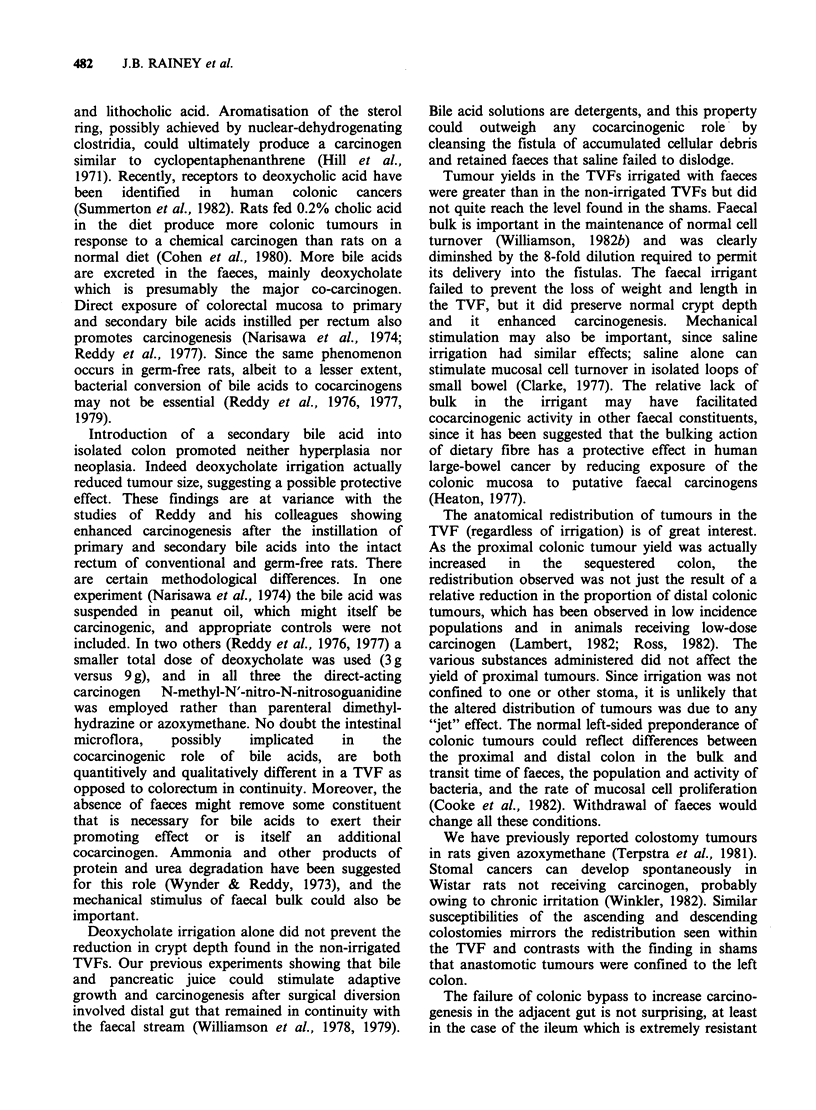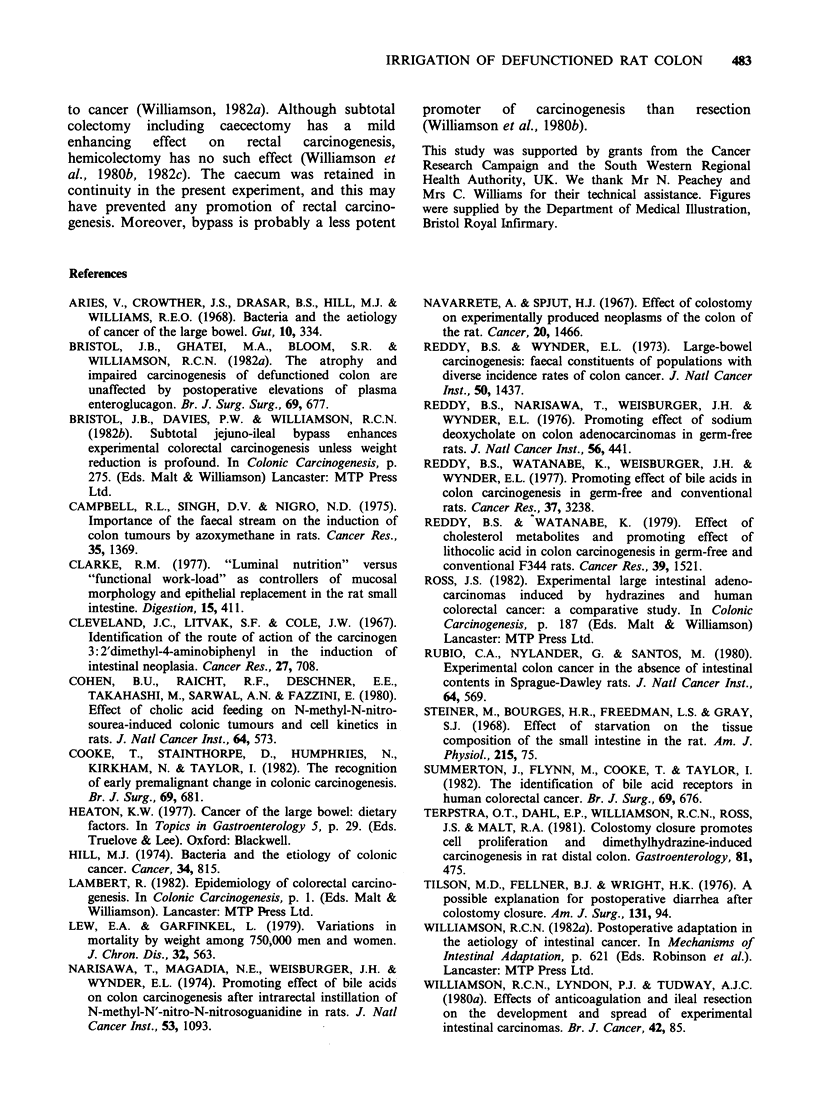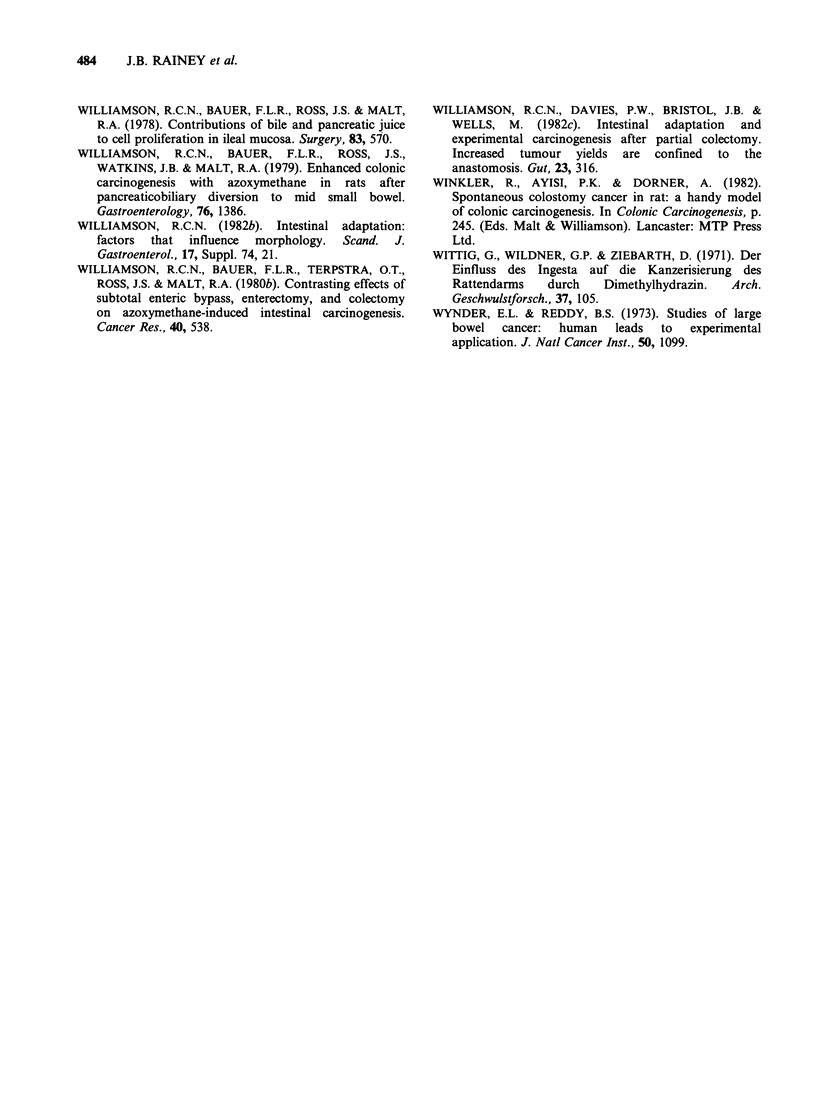# Adaptation and carcinogenesis in defunctioned rat colon: divergent effects of faeces and bile acids.

**DOI:** 10.1038/bjc.1983.220

**Published:** 1983-10

**Authors:** J. B. Rainey, P. W. Davies, J. B. Bristol, R. C. Williamson

## Abstract

Because the composition of faeces modulates colorectal carcinogenesis, promotional effects of the secondary bile salt sodium deoxycholate (SDC) were compared with those of dilute homogenised faeces (12.5% w/v) or saline alone in rat colon isolated from the faecal stream as a Thiry-Vella fistula (TVF). Each fluid was used to irrigate a group of TVFs 3 times per week for 12 weeks. Other rats had TVF without irrigation or colonic transection and reanastomosis (sham TVF). Operations followed a 6-week course of azoxymethane injections. At sacrifice 15 weeks postoperatively crypt depth and tumour yield were reduced to the same extent in both the non-irrigated TVFs and the SDC-irrigated TVFs, when compared to shams. Irrigation with faeces and saline completely restored crypt depth and partly restored tumour yields to the levels in shams. Tumours were smaller in the SDC group than in the other 4 groups. While tumours developed mainly in the left colon of shams, there was significantly more even distribution in the TVFs. Exclusion of the colon from the faecal stream leads to mucosal hypoplasia and impaired carcinogenesis. Irrigation with faeces or saline partly reverses these changes. Deoxycholate has no such effect and clearly is not co-carcinogenic in this model.


*Br. J. Cancer* (1983), **48**, 477–484

### Adaptation and carcinogenesis in defunctioned rat colon: Divergent effects of faeces and bile acids

J.B. Rainey, P.W. Davies, J.B. Bristol & R.C.N. Williamson

*University Department of Surgery, Bristol Royal Infirmary, Bristol BS2 8HW.*

**Summary** Because the composition of faeces modulates colorectal carcinogenesis, promotional effects of the secondary bile salt sodium deoxycholate (SDC) were compared with those of dilute homogenised faeces (12.5% w/v) or saline alone in rat colon isolated from the faecal stream as a Thiry-Vella fistula (TVF). Each fluid was used to irrigate a group of TVFs 3 times per week for 12 weeks. Other rats had TVF without irrigation or colonic transection and reanastomosis (sham TVF). Operations followed a 6-week course of azoxymethane injections. At sacrifice 15 weeks postoperatively crypt depth and tumour yield were reduced to the same extent in both the non-irrigated TVFs and the SDC-irrigated TVFs, when compared to shams. Irrigation with faeces and saline completely restored crypt depth and partly restored tumour yields to the levels in shams. Tumours were smaller in the SDC group than in the other 4 groups. While tumours developed mainly in the left colon of shams, there was significantly more even distribution in the TVFs. Exclusion of the colon from the faecal stream leads to mucosal hypoplasia and impaired carcinogenesis. Irrigation with faeces or saline partly reverses these changes. Deoxycholate has no such effect and clearly is not co-carcinogenic in this model.

Increased crypt-cell proliferation leads to greater numbers of intestinal tumours in susceptible animals, including man. Thus surgical shortening of the gut in rats and inflammatory bowel disease in man promote colorectal carcinogenesis (Williamson, 1982*a*). As a corollary, environmental changes that depress cell turnover might be expected to decrease carcinogenesis in the affected bowel. Starvation and interruption of normal anatomical continuity are the two most effective methods of causing intestinal hypoplasia (Steiner *et al.*, 1968; Terpstra *et al.*, 1981). Individuals who are <80% of ideal body weight may enjoy some protection against colon cancer (Lew & Garfinkel, 1979). Defunctioning proximal colostomy greatly reduces the number of cancers in the distal colon of rats exposed to azoxymethane or dimethylhydrazine, agents that reach the mucosa largely via the bloodstream (Wittig *et al.*, 1971; Campbell *et al.*, 1975). With the contact carcinogen 2,4-dimethyl-4-aminobiphenyl, which is delivered via the intestinal lumen, colostomy protects the distal colon completely (Cleveland *et al.*, 1967; Navarrete & Spjut, 1967).

Not only the presence of faeces but its precise composition affect the development of colorectal neoplasia. Diets enriched with fat and depleted of fibre increase both faecal excretion of bile acids and the number of colonic bacteria capable of further degrading secondary bile acids to potential pro-carcinogens (Reddy & Wynder, 1973; Hill, 1974). Moreover, administration of cholic acid by mouth and of deoxycholic acid per rectum promote the development of experimental tumours (Reddy *et al.*, 1976; Cohen *et al.*, 1980).

The present experiments have used loops of rat colon isolated as a Thiry-Vella fistula (TVF) to compare the local effects of saline, deoxycholic acid and dilute faeces in altering the number of tumours induced by azoxymethane. Only saline and faeces seem to reverse the atrophic and protective effects of colonic defunction.

### Materials and methods

### *Experimental animals*

One hundred and five male Sprague-Dawley rats (Olac SD, Bicester, Oxon, England) weighing 120–150 g were received into the animal house 1 week before the start of the experiment and were allocated to 1 of 5 groups (Figure 1). They were fed standard rat chow (Oxoid Breeding Diet; HC Styles & Co Ltd, Bewdley, Worcs.) and water *ad libitum*. Animal quarters were lit in alternate 12-hourly cycles. Rats were weighed weekly throughout the experiment. All animals received weekly intra-peritoneal injections of azoxymethane (Ash Stevens Inc, Detroit, Michigan, USA) 15 mg kg$^{-1}$ for 6 weeks. Each rat was submitted to operation 7–12 days after the last injection of carcinogen.

### *Surgical operations*

At laparotomy the right colon was transected 1 cm distal to its origin from the caecum, and the left colon was transected just above the pelvic brim. In

Correspondence: R.C.N. Williamson

Received 10 March 1983; accepted 22 June 1983.

© The Macmillan Press Ltd., 1983

**Figure 1** Experimental design. Number of animals at the outset and surviving to the end of the experiment (>19 weeks) are shown. TVF = Thiry-Vella fistula; SDC = sodium deoxycholate.

**Figure 2** Operations performed. (a) = sham; (b) = Thiry-Vella fistula. Shaded segments represent the sites of specimens taken for estimation of crypt depth.

groups 2–5 the stump of right colon was anastomosed to the distal cut end of left colon, thus excluding the greater part of the large bowel from the faecal stream (Figure 2*b*). This isolated loop was mobilised on the middle colic artery and its marginal branches and was brought to the surface at each end as a colonic Thiry-Vella fistula (TVF). Openings were created (5 mm diam.) in the muscle and skin of the abdominal wall on either side of the midline, and proximal and distal colostomies were established to drain the loop. In Group 1 (sham TVF), normal colonic continuity was restored by re-uniting the colon at each point of transection (Figure 2*a*).

Operations were performed under light ether anaesthesia. Continuous 6/0 silk sutures were used for intestinal anastomoses. The proximal and distal stomas were secured with interrupted 6/0 chromic catgut sutures approximating mucosa to skin. At the end of each operation 0.25 mg Vitamin K was administered i.m. to prevent the troublesome postoperative bleeding encountered in young Sprague-Dawley rats in earlier experiments (Bristol *et al.*, 1982*b*). Rats in Group 1 (sham TVF) and Group 2 (TVF alone) had no further manipulations between operation and death.

### *Irrigation of the TVF*

One week after operation the TVF was irrigated for the first time in Groups 3–5 (Figure 1). Irrigations were carried out using 5 ml plastic syringes, the hubs of which could be fitted comfortably into either stoma of rats suitably restrained by an experienced handler. An initial bolus of 5 ml N saline was administered to rats in all three groups to clear the fistula of mucus and retained faeces. It was not possible to carry out all irrigations from the same side. TVF contents (retained mucus and subsequently tumours) often produced a "ball valve" effect, making it necessary to instill volumes from each end alternately.

Once patency had been demonstrated by the appearance of the irrigant at the opposite stoma, a second bolus of irrigant was administered. Rats in Group 3 (TVF + saline) received a further 5 ml N saline. Group 4 rats (TVF + SDC) received 5 ml 0.12 M sodium deoxycholate, prepared by dissolving 50 g sodium deoxycholate (Sigma Chemical Co., St Louis, USA) in 1 litre N saline; each 5 ml aliquot contained 0.25 g (600 $\mu$mol) Na deoxycholate. Group 5 rats (TVF + faeces) received 5 ml of a 12.5% w/v suspension of rat faeces in N saline prepared by collecting and homogenising faeces from rats not receiving carcinogen. The suspension was filtered through surgical gauze to remove the larger fibres and enable delivery into the fistulas via a syringe. Each irrigant solution was administered 3 times a week (Monday, Wednesday and Friday) for 12 weeks, beginning at week 7 (Figure 1).

### *Autopsy specimens*

Rats were regularly examined for evidence of tumour development and were killed when moribund or at the end of 22 weeks. At autopsy the entire intestinal tract was excised. The following segments were thoroughly flushed with cold saline to remove all content: duodenum, jejunoileum, caecum, colon between anastomoses (or TVF) and rectum. The length of each segment was determined by suspension with a 9.5 g weight against a ruler, and the surface area of the caecum was estimated as previously described (Williamson *et al.*, 1980*a*). The weights of the liver, kidneys and spleen were also recorded. Intestinal segments were opened, and the number, size and position of all tumours were recorded. The tumours were excised, and the remaining bowel was blotted dry and weighed. All tumours were fixed in 10% formalin prior to histological processing. Subsequently 5 $\mu$m sections were prepared for staining with haematoxylin and eosin.

A 1-cm specimen of colon was excised from the middle of each TVF or from the mid transverse colon in shams, and similar histological sections were prepared. The mean crypt depth was estimated by ocular micrometry of 10 perfectly-sectioned crypts per slide.

### *Statistics*

Student's *t*-test was used for statistical analysis of the data.

### Results

### *Mortality rate*

Nine rats (8.5%) died before the end of the first postoperative week, from either haemorrhage or anaesthetic overdose. Most subsequent deaths resulted from rupture of the TVF during irrigation (9 rats), caecal volvulus around the TVF (5), or strangulated intestinal hernia. (2). Three rats with suspected burst TVFs were re-explored immediately but without success. The yields of surviving animals at the end of the experiment are given in Figure 1.

### *Body weight*

At the end of the first postoperative week the mean weight of the groups with a TVF was 5–24% lower than immediately before operation, while shams had regained their preoperative value. Thereafter, all rats gained weight steadily, but TVF rats remained a little lighter than the shams. At the end of the experiment, the mean weight of the TVF groups varied between 496 and 520 g, i.e. 91–96% of the weight of the shams ($544 \pm 16$ g, sem: $P < 0.05$).

### *Intestinal adaptation*

The mean length of the TVF in all 4 groups was $12.1 \pm 0.2$ cm (sem) and the mean weight was $2.1 \pm 0.2$ g. By contrast, the equivalent segment of colon between the anastomoses in shams was $20.0 \pm 0.6$ cm long and weighed $3.8 \pm 0.2$ g

($P>0.001$). Among the four groups of TVF rats the mean TVF length ranged from 11.4–12.9 cm and the TVF weight from 1.7–2.9 g, irrespective of irrigation. No significant differences were found between any of the groups in the weight of the duodenum, jejunoileum, caecum, liver, kidneys or spleen, nor in the surface area of the caecum or the length of the other intestinal segments.

The mean colonic crypt depth (Figure 3) in sham colons was 274 $\mu$m $\pm$ 4 (sem), compared with 242 $\pm$ 6 in the non-irrigated TVFs and 241 $\pm$ 6 in the SDC-irrigated TVFs ($P<0.001$). In those TVFs irrigated with faeces and saline, crypt depth did not differ from that in the shams (280 $\pm$ 6 and 276 $\pm$ 7).

**Figure 3** Colonic crypt depth (mean $\pm$ sem). TVF = Thiry-Vella fistula. Significance: $^*P<0.001$ *vs* other three groups.

*Intestinal tumours*

All but 2–3 rats in each group developed one or more tumours in the isolated colon (TVF) or equivalent segment of functioning colon between anastomoses (in shams). The mean number of these tumours per rat in the 4 combined TVF groups (2.7) was 25% lower than the mean number in shams (3.6) (Table I). Including those tumours arising at a stoma or colonic anastomosis increased the numbers in both TVF rats (3.0) and shams (4.3) and increased the difference to 30% ($P<0.05$). Tumour yields were lowest (64–67% of shams) in TVFs that were either not irrigated (TVF alone) or were irrigated with SDC ($P<0.05$). In rats with saline or faecal irrigation yields were still only 79–85% of those in shams, but these differences no longer attained statistical significance.

Substantial numbers of tumours arose in the duodenum, jejunoileum and rectum in each group of animals, but these numbers were not affected by creation or irrigation of a colonic TVF (Table I). In addition, one rat had a caecal tumour, one a gastric tumour and 5 had tumours of the external auditory canal. Carcinomatosis peritonei occurred in 11 rats.

The presence of a TVF altered the distribution of colonic tumours irrespective of irrigation (Figure 4).

**Figure 4** Distribution of tumours in Thiry-Vella fistulas (TVFs) or sham TVF. Each tumour in the TVF (or equivalent colon) is shown by a solid circle. Each tumour at the stoma or anastomosis is shown by a cross.

**Table I** Number of tumours per rat (mean ±s.e.) in groups with and without a colonic TVF (Thiry-Vella fistula)

| | *1. Sham TVF* | *2. TVF alone* | *3. TVF+saline* | *4. TVF+SDC* | *5. TVF+faeces* | *2–5. All TVFs* |
|---|---|---|---|---|---|---|
| Duodenum | 2.2±0.4 | 2.2±0.3 | 2.6±0.5 | 2.1±0.4 | 1.3±0.4 | 2.1±0.4 |
| Jejunoileum | 0.5±0.2 | 0.2±0.1 | 0.4±0.2 | 0.1±0.1 | 0.3±0.3 | 0.3±0.1 |
| Colon | | | | | | |
| (less stomas/anastomoses) | 3.6±0.5 | 2.4±0.4* | 2.9±0.4 | 2.3±0.4 | 3.1±0.6 | 2.7±0.2 |
| Stomas/anastomoses | 0.6±0.3 | 0.1±0.1 | 0.3±0.1 | 0.3±0.1 | 0.4±0.2 | 0.3±0.1 |
| Total Colon | 4.3±0.6 | 2.6±0.3* | 3.2±0.4 | 2.6±0.4* | 3.5±0.6 | 3.0±0.2* |
| Rectum | 1.1±0.4 | 1.9±0.3 | 1.2±0.3 | 0.9±0.3 | 1.2±0.3 | 1.3±0.2 |

SDC = Sodium deoxycholate.
Significance *versus* shams.
* = $P<0.05$.

In sham rats 41 of 52 tumours (81%) arose within the distal half of the colon, whereas in TVF rats (groups 2–5) tumours were evenly distributed between the proximal (49%) and distal (51%) halves of the isolated colon. The yield of tumours in the proximal colon was 1.4±0.1 in all TVFs compared with 0.7±0.3 in the equivalent segment in shams ($P<0.05$). No differences were observed between the non-irrigated and irrigated TVFs. Similarly, in TVF rats, tumours were distributed almost equally between the right stoma (9 tumours) and the left stoma (8), while in shams anastomotic tumours (9) were confined to the left colon.

The mean diameter of tumours found in the TVFs irrigated with sodiumdeoxycholate (2.6±0.3 mm) was 41–51% less than the diameter of colonic tumours in the other 4 groups ($P<0.01$). There were no other significant differences, however, and creating a TVF alone did not reduce tumour size.

Adenocarcinoma was the commonest histological type (80%), varying from carcinoma-in-situ to invasive cancer. Mucinous (colloid) adenocarcinomas (7%), characterised by the presence of "signet-ring" cells, were detected mostly in the duodenum and were all deeply invasive. Benign adenomas and hyperplastic polyps accounted for 13% of tumours detected. No differences in tumour histology were observed between the groups.

### Discussion

Defunctioned colon develops fewer tumours than colon remaining in continuity. This finding can hardly be explained by the minor decrease in body weight found in the TVF rats, as we have shown in a previous study that 85% jejunoileal bypass enhances colonic carcinogenesis despite a 40% reduction in body weight (Bristol *et al.*, 1982*b*). Our results are consistent with the findings of other workers (Wittig *et al.*, 1971; Campbell *et al.*, 1975) who have ascribed the protective effect of a defunctioning proximal colostomy to an altered population of bile acids and bacteria in the excluded distal colorectum. Our preliminary findings that neither resection nor bypass of the small intestine affect carcinogenesis in bypassed colon suggest that hormones are of secondary importance to a diversion of the faecal stream (Bristol *et al.*, 1982*a*). Rubio *et al.* (1980) in a similar experiment found no reduction in dimethylhydrazine-induced tumours in a small colonic TVF, but the number of rats was small and no control group was included.

Mucosal hypoplasia in the bypassed colon is indicated by shortening of the crypts and by the reduced weights and lengths of the TVFs; presumably this phenomenon accounts for the reduced susceptibility to carcinogenesis. There was a close correlation between tumour yield and crypt depth, each being reduced in the TVF (with or without deoxycholate) but indistinguishable from values in intact colon after irrigation of the TVF with saline or faeces. Other studies confirm that mucosal atrophy develops rapidly distal to the site of a proximal colostomy but is precisely reversed soon after continuity of the bowel is restored (Tilson *et al.*, 1976; Terpstra *et al.*, 1981).

Our results may shed some light on the role of various faecal constituents in colorectal carcinogenesis. Clearly, deoxycholate was not cocarcinogenic in this model, despite strong epidemiological and experimental evidence to support such a role for bile acids. According to Aries *et al.* (1969), their increased faecal excretion could well be the link between high-fat diets and susceptibility to cancer, especially after bacterial degradation to the secondary forms, deoxycholic and lithocholic acid. Aromatisation of the sterol ring, possibly achieved by nuclear-dehydrogenating clostridia, could ultimately produce a carcinogen similar to cyclopentaphenanthrene (Hill *et al.*, 1971). Recently, receptors to deoxycholic acid have been identified in human colonic cancers (Summerton *et al.*, 1982). Rats fed 0.2% cholic acid in the diet produce more colonic tumours in response to a chemical carcinogen than rats on a normal diet (Cohen *et al.*, 1980). More bile acids are excreted in the faeces, mainly deoxycholate which is presumably the major co-carcinogen. Direct exposure of colorectal mucosa to primary and secondary bile acids instilled per rectum also promotes carcinogenesis (Narisawa *et al.*, 1974; Reddy *et al.*, 1977). Since the same phenomenon occurs in germ-free rats, albeit to a lesser extent, bacterial conversion of bile acids to cocarcinogens may not be essential (Reddy *et al.*, 1976, 1977, 1979).

Introduction of a secondary bile acid into isolated colon promoted neither hyperplasia nor neoplasia. Indeed deoxycholate irrigation actually reduced tumour size, suggesting a possible protective effect. These findings are at variance with the studies of Reddy and his colleagues showing enhanced carcinogenesis after the instillation of primary and secondary bile acids into the intact rectum of conventional and germ-free rats. There are certain methodological differences. In one experiment (Narisawa *et al.*, 1974) the bile acid was suspended in peanut oil, which might itself be carcinogenic, and appropriate controls were not included. In two others (Reddy *et al.*, 1976, 1977) a smaller total dose of deoxycholate was used (3 g versus 9 g), and in all three the direct-acting carcinogen N-methyl-N′-nitro-N-nitrosoguanidine was employed rather than parenteral dimethylhydrazine or azoxymethane. No doubt the intestinal microflora, possibly implicated in the cocarcinogenic role of bile acids, are both quantitively and qualitatively different in a TVF as opposed to colorectum in continuity. Moreover, the absence of faeces might remove some constituent that is necessary for bile acids to exert their promoting effect or is itself an additional cocarcinogen. Ammonia and other products of protein and urea degradation have been suggested for this role (Wynder & Reddy, 1973), and the mechanical stimulus of faecal bulk could also be important.

Deoxycholate irrigation alone did not prevent the reduction in crypt depth found in the non-irrigated TVFs. Our previous experiments showing that bile and pancreatic juice could stimulate adaptive growth and carcinogenesis after surgical diversion involved distal gut that remained in continuity with the faecal stream (Williamson *et al.*, 1978, 1979). Bile acid solutions are detergents, and this property could outweigh any cocarcinogenic role by cleansing the fistula of accumulated cellular debris and retained faeces that saline failed to dislodge.

Tumour yields in the TVFs irrigated with faeces were greater than in the non-irrigated TVFs but did not quite reach the level found in the shams. Faecal bulk is important in the maintenance of normal cell turnover (Williamson, 1982*b*) and was clearly diminshed by the 8-fold dilution required to permit its delivery into the fistulas. The faecal irrigant failed to prevent the loss of weight and length in the TVF, but it did preserve normal crypt depth and it enhanced carcinogenesis. Mechanical stimulation may also be important, since saline irrigation had similar effects; saline alone can stimulate mucosal cell turnover in isolated loops of small bowel (Clarke, 1977). The relative lack of bulk in the irrigant may have facilitated cocarcinogenic activity in other faecal constituents, since it has been suggested that the bulking action of dietary fibre has a protective effect in human large-bowel cancer by reducing exposure of the colonic mucosa to putative faecal carcinogens (Heaton, 1977).

The anatomical redistribution of tumours in the TVF (regardless of irrigation) is of great interest. As the proximal colonic tumour yield was actually increased in the sequestered colon, the redistribution observed was not just the result of a relative reduction in the proportion of distal colonic tumours, which has been observed in low incidence populations and in animals receiving low-dose carcinogen (Lambert, 1982; Ross, 1982). The various substances administered did not affect the yield of proximal tumours. Since irrigation was not confined to one or other stoma, it is unlikely that the altered distribution of tumours was due to any "jet" effect. The normal left-sided preponderance of colonic tumours could reflect differences between the proximal and distal colon in the bulk and transit time of faeces, the population and activity of bacteria, and the rate of mucosal cell proliferation (Cooke *et al.*, 1982). Withdrawal of faeces would change all these conditions.

We have previously reported colostomy tumours in rats given azoxymethane (Terpstra *et al.*, 1981). Stomal cancers can develop spontaneously in Wistar rats not receiving carcinogen, probably owing to chronic irritation (Winkler, 1982). Similar susceptibilities of the ascending and descending colostomies mirrors the redistribution seen within the TVF and contrasts with the finding in shams that anastomotic tumours were confined to the left colon.

The failure of colonic bypass to increase carcinogenesis in the adjacent gut is not surprising, at least in the case of the ileum which is extremely resistant to cancer (Williamson, 1982*a*). Although subtotal colectomy including caecectomy has a mild enhancing effect on rectal carcinogenesis, hemicolectomy has no such effect (Williamson *et al.*, 1980*b*, 1982*c*). The caecum was retained in continuity in the present experiment, and this may have prevented any promotion of rectal carcinogenesis. Moreover, bypass is probably a less potent promoter of carcinogenesis than resection (Williamson *et al.*, 1980*b*).

This study was supported by grants from the Cancer Research Campaign and the South Western Regional Health Authority, UK. We thank Mr N. Peachey and Mrs C. Williams for their technical assistance. Figures were supplied by the Department of Medical Illustration, Bristol Royal Infirmary.

### References

ARIES, V., CROWTHER, J.S., DRASAR, B.S., HILL, M.J. & WILLIAMS, R.E.O. (1968). Bacteria and the aetiology of cancer of the large bowel. *Gut*, **10**, 334.

BRISTOL, J.B., GHATEI, M.A., BLOOM, S.R. & WILLIAMSON, R.C.N. (1982*a*). The atrophy and impaired carcinogenesis of defunctioned colon are unaffected by postoperative elevations of plasma enteroglucagon. *Br. J. Surg. Surg.*, **69**, 677.

BRISTOL, J.B., DAVIES, P.W. & WILLIAMSON, R.C.N. (1982*b*). Subtotal jejuno-ileal bypass enhances experimental colorectal carcinogenesis unless weight reduction is profound. In *Colonic Carcinogenesis*, p. 275. (Eds. Malt & Williamson) Lancaster: MTP Press Ltd.

CAMPBELL, R.L., SINGH, D.V. & NIGRO, N.D. (1975). Importance of the faecal stream on the induction of colon tumours by azoxymethane in rats. *Cancer Res.*, **35**, 1369.

CLARKE, R.M. (1977). "Luminal nutrition" versus "functional work-load" as controllers of mucosal morphology and epithelial replacement in the rat small intestine. *Digestion*, **15**, 411.

CLEVELAND, J.C., LITVAK, S.F. & COLE, J.W. (1967). Identification of the route of action of the carcinogen 3:2′dimethyl-4-aminobiphenyl in the induction of intestinal neoplasia. *Cancer Res.*, **27**, 708.

COHEN, B.U., RAICHT, R.F., DESCHNER, E.E., TAKAHASHI, M., SARWAL, A.N. & FAZZINI, E. (1980). Effect of cholic acid feeding on N-methyl-N-nitrosourea-induced colonic tumours and cell kinetics in rats. *J. Natl Cancer Inst.*, **64**, 573.

COOKE, T., STAINTHORPE, D., HUMPHRIES, N., KIRKHAM, N. & TAYLOR, I. (1982). The recognition of early premalignant change in colonic carcinogenesis. *Br. J. Surg.*, **69**, 681.

HEATON, K.W. (1977). Cancer of the large bowel: dietary factors. In *Topics in Gastroenterology 5*, p. 29. (Eds. Truelove & Lee). Oxford: Blackwell.

HILL, M.J. (1974). Bacteria and the etiology of colonic cancer. *Cancer*, **34**, 815.

LAMBERT, R. (1982). Epidemiology of colorectal carcinogenesis. In *Colonic Carcinogenesis*, p. 1. (Eds. Malt & Williamson). Lancaster: MTP Press Ltd.

LEW, E.A. & GARFINKEL, L. (1979). Variations in mortality by weight among 750,000 men and women. *J. Chron. Dis.*, **32**, 563.

NARISAWA, T., MAGADIA, N.E., WEISBURGER, J.H. & WYNDER, E.L. (1974). Promoting effect of bile acids on colon carcinogenesis after intrarectal instillation of N-methyl-N′-nitro-N-nitrosoguanidine in rats. *J. Natl Cancer Inst.*, **53**, 1093.

NAVARRETE, A. & SPJUT, H.J. (1967). Effect of colostomy on experimentally produced neoplasms of the colon of the rat. *Cancer*, **20**, 1466.

REDDY, B.S. & WYNDER, E.L. (1973). Large-bowel carcinogenesis: faecal constituents of populations with diverse incidence rates of colon cancer. *J. Natl Cancer Inst.*, **50**, 1437.

REDDY, B.S., NARISAWA, T., WEISBURGER, J.H. & WYNDER, E.L. (1976). Promoting effect of sodium deoxycholate on colon adenocarcinomas in germ-free rats. *J. Natl Cancer Inst.*, **56**, 441.

REDDY, B.S., WATANABE, K., WEISBURGER, J.H. & WYNDER, E.L. (1977). Promoting effect of bile acids in colon carcinogenesis in germ-free and conventional rats. *Cancer Res.*, **37**, 3238.

REDDY, B.S. & WATANABE, K. (1979). Effect of cholesterol metabolites and promoting effect of lithocolic acid in colon carcinogenesis in germ-free and conventional F344 rats. *Cancer Res.*, **39**, 1521.

ROSS, J.S. (1982). Experimental large intestinal adenocarcinomas induced by hydrazines and human colorectal cancer: a comparative study. In *Colonic Carcinogenesis*, p. 187 (Eds. Malt & Williamson) Lancaster: MTP Press Ltd.

RUBIO, C.A., NYLANDER, G. & SANTOS, M. (1980). Experimental colon cancer in the absence of intestinal contents in Sprague-Dawley rats. *J. Natl Cancer Inst.*, **64**, 569.

STEINER, M., BOURGES, H.R., FREEDMAN, L.S. & GRAY, S.J. (1968). Effect of starvation on the tissue composition of the small intestine in the rat. *Am. J. Physiol.*, **215**, 75.

SUMMERTON, J., FLYNN, M., COOKE, T. & TAYLOR, I. (1982). The identification of bile acid receptors in human colorectal cancer. *Br. J. Surg.*, **69**, 676.

TERPSTRA, O.T., DAHL, E.P., WILLIAMSON, R.C.N., ROSS, J.S. & MALT, R.A. (1981). Colostomy closure promotes cell proliferation and dimethylhydrazine-induced carcinogenesis in rat distal colon. *Gastroenterology*, **81**, 475.

TILSON, M.D., FELLNER, B.J. & WRIGHT, H.K. (1976). A possible explanation for postoperative diarrhea after colostomy closure. *Am. J. Surg.*, **131**, 94.

WILLIAMSON, R.C.N. (1982*a*). Postoperative adaptation in the aetiology of intestinal cancer. In *Mechanisms of Intestinal Adaptation*, p. 621 (Eds. Robinson *et al.*). Lancaster: MTP Press Ltd.

WILLIAMSON, R.C.N., LYNDON, P.J. & TUDWAY, A.J.C. (1980*a*). Effects of anticoagulation and ileal resection on the development and spread of experimental intestinal carcinomas. *Br. J. Cancer*, **42**, 85.

WILLIAMSON, R.C.N., BAUER, F.L.R., ROSS, J.S. & MALT, R.A. (1978). Contributions of bile and pancreatic juice to cell proliferation in ileal mucosa. *Surgery*, **83**, 570.

WILLIAMSON, R.C.N., BAUER, F.L.R., ROSS, J.S., WATKINS, J.B. & MALT, R.A. (1979). Enhanced colonic carcinogenesis with azoxymethane in rats after pancreaticobiliary diversion to mid small bowel. *Gastroenterology*, **76**, 1386.

WILLIAMSON, R.C.N. (1982*b*). Intestinal adaptation: factors that influence morphology. *Scand. J. Gastroenterol.*, **17**, Suppl. 74, 21.

WILLIAMSON, R.C.N., BAUER, F.L.R., TERPSTRA, O.T., ROSS, J.S. & MALT, R.A. (1980*b*). Contrasting effects of subtotal enteric bypass, enterectomy, and colectomy on azoxymethane-induced intestinal carcinogenesis. *Cancer Res.*, **40**, 538.

WILLIAMSON, R.C.N., DAVIES, P.W., BRISTOL, J.B. & WELLS, M. (1982*c*). Intestinal adaptation and experimental carcinogenesis after partial colectomy. Increased tumour yields are confined to the anastomosis. *Gut*, **23**, 316.

WINKLER, R., AYISI, P.K. & DORNER, A. (1982). Spontaneous colostomy cancer in rat: a handy model of colonic carcinogenesis. In *Colonic Carcinogenesis*, p. 245. (Eds. Malt & Williamson). Lancaster: MTP Press Ltd.

WITTIG, G., WILDNER, G.P. & ZIEBARTH, D. (1971). Der Einfluss des Ingesta auf die Kanzerisierung des Rattendarms durch Dimethylhydrazin. *Arch. Geschwulstforsch.*, **37**, 105.

WYNDER, E.L. & REDDY, B.S. (1973). Studies of large bowel cancer: human leads to experimental application. *J. Natl Cancer Inst.*, **50**, 1099.